# Comparison of chromosomal status in reserved multiple displacement amplification products of embryos that resulted in miscarriages or live births: a blinded, nonselection case–control study

**DOI:** 10.1186/s12920-022-01187-y

**Published:** 2022-02-23

**Authors:** Guoxia Yang, Yan Xu, Yanhong Zeng, Jing Guo, Jiafu Pan, Canquan Zhou, Yanwen Xu

**Affiliations:** 1 The First Affiliated Hospital, Sun Yat-Sen University, Guangzhou, Guangdong People’s Republic of China; 2 Guangdong Provincial Key Laboratory of Reproductive Medicine, The First Affiliated Hospital, Sun Yat-Sen University, Guangzhou, Guangdong People’s Republic of China; 3 Reproductive Medical Center, the First Affiliated Hospital, Sun Yat-Sen University, Zhongshan 2nd Road, Guangzhou, Guangdong People’s Republic of China

**Keywords:** Chromosomal abnormalities, Preimplantation genetic testing for monogenic disorders (PGT-Ms), Preimplantation genetic testing for aneuploidy screening (PGT-A), Mosaicism

## Abstract

**Objective:**

To analyze chromosomal status in reserved multiple displacement amplification (MDA) products of embryos that result in miscarriages or live births.

**Methods:**

Patients who underwent preimplantation genetic testing for monogenic disorders (PGT-Ms) without aneuploidy screening were included. The case group included 28 cycles that resulted in miscarriages. Controls included 56 cycles with live births. Comprehensive chromosomal screening (CCS) using next-generation sequencing (NGS) was performed on reserved MDA products from previous blastocyst trophectoderm biopsies. The incidence and type of chromosomal abnormalities in embryos resulting in miscarriages or live births were analyzed.

**Results:**

Of 28 embryos resulting in miscarriages in the case group, the rate of chromosomal abnormalities was 53.6%, which was significantly greater than 14.3% for those resulting in live births in control group (*P* < 0.001). Whole-chromosome aneuploidy was not found in the control group but was noted in 25.0% of embryos in the case group. Although the rates of segmental abnormality and mosaicism were also greater in the case group, no significant differences were detected. One chaotic embryo in the control group progressed to live birth.

**Conclusion:**

Chromosomal abnormalities were the main reason leading to early pregnancy loss. However, abnormalities, such as segmental aneuploidy and mosaicism, should be managed cautiously, considering their undermined reproductive potential.

## Background

Due to errors predominantly in meiosis, aneuploidy is considered to be the main cause of failed implantation, early miscarriages, and congenital birth defects [1, 2]. Increasing maternal age is a powerful contributor to the occurrence of aneuploidy [3]. In recent years, the application of preimplantation genetic testing for aneuploidy (PGT-A) has been increasingly used for the purpose of selecting a euploid embryo for transfer, thus improving clinical outcomes. The advantages of PGT-A include an increase in the live birth rate, a decrease in the miscarriage rate, and a shortened time interval to pregnancy [4, 5].

Next-generation sequencing (NGS) is currently applied as an effective technique for the analysis of copy number variation in few cell biopsies during PGT-A. In particular, the NGS platform was demonstrated to be useful to detect a broader dynamic range of chromosomal aberrations with higher resolution and sensitivity. Thus, this platform is capable of detecting mosaicism and subchromosomal abnormalities, such as segmental duplications and deletions affecting regions greater than 5–10 Mb [6–8].

Although NGS and trophectoderm (TE) biopsy have become the preferred technique for PGT-A, it must be noted that technical noise introduced by whole genome amplification and NGS may cause false-positive results [9]. Furthermore, biopsied samples with 5–10 TE cells may be insufficient for reflecting the true genetic status of the embryo, especially for segmental aneuploidy and mosaicism. A recent study showed a high reliability of TE biopsy for euploids and aneuploids (greater than 95%), but the technique was less reliable in mosaic embryos (35.2%) [10]. Another study found that whole-chromosome aneuploidy in TE biopsy is predictive of aneuploidy in the inner cell mass (ICM) in 96.8% (90/93), but this value decreased to 42.9% for segmental aneuploidy [11]. Moreover, 30.8% (12/39) of embryos labeled segmental aneuploidies resulted in sustained pregnancies or healthy live births [12]. On the other hand, false negative errors also existed, although they were estimated to be less than 4% [13].

Here, we aimed to compare the incidence and type of chromosomal abnormalities of embryos transferred blindly that resulted in miscarriages with those resulting in live births by a blinded, nonselection, matched case–control study with the goal of uncovering the role of chromosomal abnormalities in miscarriage. In addition, chromosomal abnormalities in embryos that resulted in live births were analyzed, which could only be explained by false-positive diagnosis in these reproductive competent embryos.

## Materials and methods

### Study population

This was a blinded, nonselection, matched case–control study conducted at the Reproductive Medicine Center of the First Affiliated Hospital of Sun Yat-sen University. All cases were subject to PGT-M by gap polymerase chain reaction (PCR) or nested PCR after whole genome amplification using multiple displacement amplification (MDA), as described in our previously published papers [14, 15]. Indications for PCR-based mutation detection in these embryos were α- or β-thalassemia. Aneuploidy screening was not performed before frozen/thawed embryo transfer (FET). Pregnancies after FETs that resulted in early spontaneous miscarriage were identified from January 2019 to June 2020. Patients were excluded if they had a known history of recurrent pregnancy loss (two or more clinical miscarriages), endocrine disorder, severe intrauterine adhesions, and endometrial polyps. A total of 39 cases met the inclusion criteria during the study period, of which 2 were excluded for cleavage stage biopsy, 4 due to miscarriage in the 2nd trimester of pregnancy, and 5 due to the poor amplification effect of the previous DNA products. Finally, 28 patients comprised the case group (n = 28). During the same period, 386 cases of PGT-M achieved live births. After matching the date of oocyte retrieval and maternal age with the case group, 56 cases that fulfilled the inclusion criteria were selected as the control group.

### Oocyte retrieval, embryo culture, and biopsy

All patients underwent ovarian stimulation and oocyte retrieval according to our standard protocols. Intracytoplasmic sperm injection (ICSI) was used in all cases for insemination. Embryos were cultured in separate droplets using standard incubation conditions (5% O_2_ and 6% CO_2_). Embryo morphology was graded using Gardner’s criteria [16]. Blastocysts with dense cellular inner cell mass (ICMs) and TEs were considered to be of highest morphologic quality. TE biopsy was performed on Day 5 or Day 6 after oocyte retrieval and vitrified separately just after the biopsy, as described in our previous study [17].

### Multiple displacement amplification (MDA) and PCR analysis

The biopsied TE cells transferred to lysis buffer were subjected to whole-genome amplification (WGA) using the MDA approach (REPLI-g Single Cell Kit; QIAGEN, Inc.) as previously described [14, 15]. Then, the MDA products were used as templates in PCR analysis of α-thalassemia or β-thalassemia as shown in our published paper [15]. The remaining MDA products were frozen in -80℃. PCR products were then analyzed on an ABI 3100 Advant genetic analyzer for α-thalassemia or underwent reverse dot hybridization for β-thalassemia. Single embryo transfers were performed following warming of the cryopreserved blastocysts that were not affected with monogenetic disease as determined by PCR, including the normal homozygous and heterozygous blastocysts. Spontaneous miscarriage described a pregnancy in which an intrauterine gestational sac was visualized, but the pregnancy did not progress or was spontaneously lost before13 weeks.

### Next-generation sequencing (NGS)

Reserved MDA products were reanalyzed by NGS (Illumina) for comprehensive chromosomal screening. The span between MDA and reanalysis by NGS was within 3 years. Karyotype profiles were scored independently by two analysts using MiSeq Reporter software (Illumina), which depicts the copy number variation (CNV) for each chromosome in a sample. CNV values less than 1.20 or greater than 2.80 were labeled monosomy or trisomy; CNV values between 1.80 and 2.20 were considered euploid; and aneuploidies with CNV values between 1.20 and 1.80 or between 2.20 and 2.80 were considered mosaicism. Chaotic embryos were defined as those showing a complex pattern of aneuploidies involving more than six chromosomes [18]. Genomic referrence for sequence alignment is hg19. All personnel analyzing the biopsies were blinded to the clinical outcomes.

### Outcome measures

The primary outcome was the incidence and type of chromosomal abnormalities detected by NGS in the two groups.

### Statistical analyses

Data are presented as the mean ± SD or median for continuous variables and as percentage for categorical variables. Continuous variables were analyzed using a *t* test or nonparametric test. Categorical data were compared with the chi-square test or Fisher's exact test with 95% confidence intervals. *P* values of < 0.05 were considered statistically significant. Data analysis was performed using the statistical software SPSS 22.0.

## Results

### General characteristics of study participants

The general characteristics of the study participants are shown in Table 1. There were no significant differences between the two groups with regard to the average maternal age on oocyte retrieval day, body mass index (BMI), gravidity and parity, number of miscarriages, basal levels of FSH and E_2_, E_2_ level on HCG day, number of retrieved oocytes, number of good-quality embryos, day of blastocyst transfer, and controlled ovarian stimulation (COS) protocols.

**Table 1:** Baseline characteristics of patients with miscarriage and live birth controls

Characteristics	Miscarriage cases (n = 28)	Live-birth controls (n = 56)	*P* value
Maternal age at oocyte retrieval, y	29.6 ± 3.7	29.6 ± 3.2	0.945
Maternal BMI, kg/m^2^	21.8 ± 3.1	21.3 ± 2.7	0.421
Gravidity	1.0 (0–4)	2.0 (0–4)	0.194^a^
Parity	0.0 (0–1)	0.0 (0–2)	0.932^a^
No. of previous miscarriages, n	1.0 (0–4)	2.0 (0–4)	0.095^a^
Basal E_2_ level, pg/ml	29.2 ± 9.4	29.0 ± 13.5	0.940
Basal FSH level, IU/ml	5.6 ± 0.9	5.5 ± 1.3	0.726
E_2_ level on HCG day, pg/ml	3141 ± 1387.9	3127 ± 1110.1	0.962
No. of retrieved oocytes	21.1 ± 10.7	19.3 ± 7.8	0.390
No. of good-quality blastocysts	6.8 ± 4.6	6.6 ± 3.4	0.809
No. of embryos available to transfer	6.9 ± 4.0	6.5 ± 2.8	0.618
Blastocyst transfer			0.380^b^
D5	19 (67.9)	43 (76.8)	
D6	9 (32.1)	13 (23.2)	
COS protocol			0.071^b^
Long agonist	4 (14.3)	19 (33.9)	
Short agonist	6 (21.4)	15 (26.8)	
Antagonist	18 (64.3)	22 (39.3)	

Values are presented as mean ± SD, n (range), or n (%)

*BMI* body mass index, *E*_*2*_ estradiol, *FSH* follicle-stimulating hormone, *HCG* human chorionic gonadotropin, *COS* controlled ovarian stimulation

^a^Mann–Whitney U test

^b^Chi-square test

### Chromosomal abnormalities in two groups

The chromosomal abnormality pattern is presented in Table 2. In the case group, 53.6% (15/28) of embryos were subject to chromosomal abnormality testing by NGS, which was significantly higher than 14.3% in the control group (*P* < 0.001), with an OR of 2.262 (95% CI 1.273–4.019). Furthermore, no whole-chromosome aneuploidy was found in the control group but was noted in 25.0% in the case group (25.0% vs. 0%; *P* < 0.001).

**Table 2:** Results of reanalysis of MDA products with next-generation sequencing

NGS results	Miscarriage cases (n = 28)	Live birth controls (n = 56)	*P* value^a^	OR (95%CI)
Euploid	13 (46.4)	48 (85.7)	< 0.001	0.327 (0.185–0.576)
Whole-chromosome aneuploid	7 (25.0)	0	< 0.001	
Mosaic	5 (17.9)	5 (8.9)	0.404	1.378 (0.728–2.610)
Segmental abnormality	3 (10.7)	2 (3.6)	0.327	1.709 (0.578–5.052)
Chaotic	0	1 (1.8)	0.003	

Values are presented as n (%)

*MDA* multiple displacement amplification, *NGS* next-generation sequencing

^a^Fisher's exact test

**Fig. 1:**
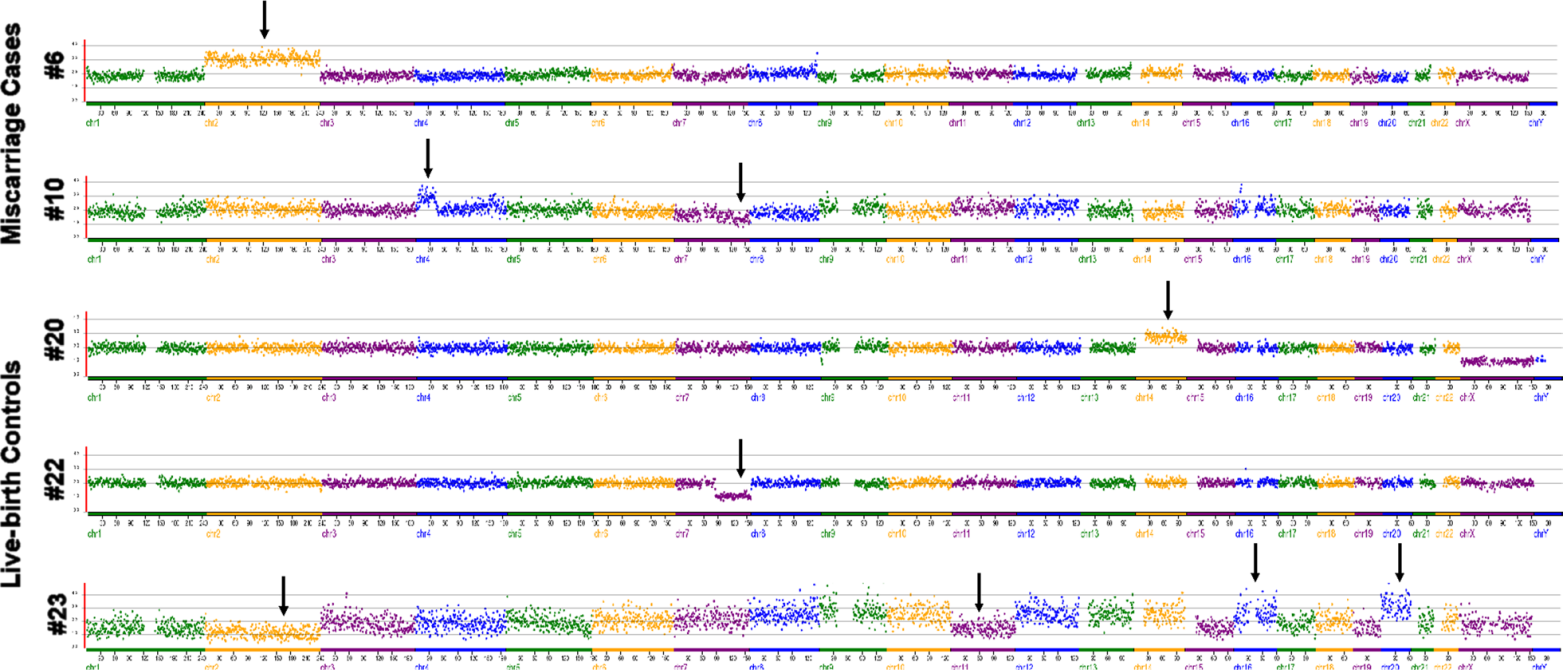
Examples of chromosomal abnormalities detected by NGS in miscarriage cases and live birth controls. Blastocyst #6 was identified as having trisomy 2. Blastocyst #10 was identified as having both a duplication on ch.4p16.1-p13 and mosaicism with partial deletion on 7q21.13-qter (59%). Blastocyst #20 was identified as having whole chromosome mosaicism on ch.14 (75%). Blastocyst #22 exhibited segmental aneuploidy with deletion of ch.7q21.11-q36.3. Blastocyst #23 demonstrated a complex pattern of aneuploidies with monosomy 2 and 11 and trisomy 16 and 20

All abnormal NGS results are shown in Table 3. Mosaicism was detected in 5 out of 28 cases, which was two-fold greater than that in the control group (17.9% vs*.* 8.9%). However, the difference did not reach significance (95% CI 0.728–2.610; *P* = 0.404). Importantly, high level of segmental mosaicism was observed in three control cases, 54%, 68%, and 75%, respectively. In total, there were 3 and 2 segmental abnormalities in the case group and control group, respectively. The occurrence of segmental abnormalities in the case group was also higher (10.7% vs. 3.6%), but no significant difference was found. The segmental length in the 2 cases of the control group was 74.8 Mb and 7.3 Mb, separately. In addition, one chaotic embryo in the control group progressed to live birth. Examples of chromosomal abnormalities detected by NGS in miscarriage cases and live birth controls are shown in Fig. 1.

**Table 3:** Abnormal NGS results of reserved MDA products

Blastocyst no.	Patient age (y)	NGS results	Segmental length (Mb)	Percentage of mosaicism (%)	Pregnancy outcome
1	32	47, XY, + 21			Miscarriage
2	35	47, XY, + 21			Miscarriage
3	27	47, XX, + 22			Miscarriage
4	28	47, XY, + 22			Miscarriage
5	37	47, XY, + 22			Miscarriage
6	34	47, XX, + 2			Miscarriage
7	28	45, XX, -14, dup(15)^*^		57	Miscarriage
8	29	46, XX, dup(2)(p25.3p24.3)(10001–13787489)	13.8		Miscarriage
9	28	46, XX, dup(9)(q21.11q34.3)(70700080–141017812)	70.3		Miscarriage
10	27	46, XX, dup(4)(p16.1p13)(8837564–43148033), del(7)(q21.13q36.3)^*^(89919065–159127103)	34.3, 69.2	59	Miscarriage
11	27	46, XX, del(21)(q21.3q22.3)^*^(29581247–47924389)	18.3	63	Miscarriage
12	27	46, XX, del(18)^*^		43	Miscarriage
13	27	46, XY, del(4)(q21.21q35.2)^*^(82193244–191016503)	108.8	35	Miscarriage
14	33	46, XY, del(12)(q23.2q24.33)^*^(103339018–133719849)	30.4	47	Miscarriage
15	31	46, XX, dup(1) ^*^, dup(13)^*^		60, 52	Miscarriage
16	29	46, XX, del(11)(p15.4p15.5)^*^(9786610–175991)	9.6	68	Live birth
17	32	46, XY, dup(3)(p21.1q22.3)^*^(54243468–138560081), del(3)(q26.1q29)^*^(164176163–197849761)	84.3, 33.7	35, 30	Live birth
18	27	46, XY, dup(Y)^*^		41	Live birth
19	28	46, XX, del(4)^*^		54	Live birth
20	24	46, XY, dup(14) ^*^		75	Live birth
21	27	46, XX, del(17)(q25.1q25.3)(73886680–81195210)	7.3		Live birth
22	29	46, XX, del(7)(q21.11q36.3)(84338306–159127103)	74.8		Live birth
23	27	46, XX, − 2, − 11, + 16, + 20			Live birth

* Mosaic, *dup* duplication, *del* deletion. Genomic referrence for sequence alignment is hg19

## Discussion

In this study, the rate of chromosomal abnormality was significantly higher in embryos resulting in miscarriages. No viable pregnancy was achieved in embryos with whole-chromosome aneuploidies; however, embryos with other abnormalities may have a chance to live births.

It is well accepted that aneuploidy is one of the main causes of early spontaneous miscarriage. Our previous study showed that PGT-A can reduce the miscarriage rate of young women by 73.45% in the first FET cycles [5]. In this study, compared with embryos with live births, the rate of chromosomal abnormalities in embryos resulting in miscarriages was significantly higher. Our observation was consistent with the result of a previous study showing a 51.82% abnormal chromosomal rate in a cohort of 164 spontaneous miscarriages with no previous aneuploidy screening [1]. In addition, all embryos with whole-chromosome aneuploid belonged to the case group. This finding is consistent with a recent report showing that no embryos (0/102) diagnosed with whole-chromosome aneuploid progressed to delivery [12], and a recent study analyzing 61 full aneuploid embryo transfers that revealed 0% sustained implantation [19]. Considering these data, the positive predictive value of whole-chromosome aneuploidy was very high.

Question remains on the developmental potential of embryos with segmental abnormalities or mosaicism. NGS-based studies have revealed that the incidence of segmental abnormalities in human blastocysts is 2.4–7.5% [20, 21]. In the study of Girardi et al., approximately 70% of segmental aneuploidies were of mitotic origin [20], and the positive predictive value toward ICM configuration was 70.8%, which is significantly lower than 97.18% of whole chromosome aneuploidies. An estimated 32% of embryos originated from meiotic error and presented throughout the whole blastocyst [20], which is associated with implantation failure or miscarriage. Navratil et al*.* also compared the results of TE biopsies to entire embryos. They found concordance between original TE biopsies and entire embryos only in 14 (45.2%) out of 31 for segmental abnormalities[22]. In the present study, 3 and 2 segmental aneuploidies were detected in the case group and the control group, respectively, and the segmental length in the 2 cases of the control group was 74.8 Mb and 7.3 Mb, separately. Supposing that these two segmental aneuploidies were not confirmed in a second biopsy, the likelihood of euploid ICM increased to 89.5%, according to a risk stratification model developed by Girardi et al. [20]. Our results confirmed the low predictive value of segmental aneuploidies. In our routine practice, these two embryos with segmental aneuploidies in the control group will not have the chance to be transferred.

Chromosomal mosaicism is defined as the presence of two or more distinct cell lines within one embryo, and the most common type is mosaic of euploid and aneuploid cells [13]. Mosaicism is derived from mitotic errors during the first cleavage divisions. The load of abnormal cells may affect the reproductive competence of embryos [23]. Embryos with a high number of aneuploid cells ultimately result in early arrest, as demonstrated in a mouse model of chromosomal mosaicism [24]. Several investigations have shown that mosaic embryos have a significantly poorer implantation and live birth rate than euploids [25]. Of those, embryos with a high-level of abnormal cells [26] or complex segmental mosaic [27] performed worse.

TE biopsies diagnosed as euploid by array comparative genomic hybridization (aCGH) were reanalyzed by NGS in the study by Maxwell et al*.*. Specifically, 31.6% were mosaics in miscarriage group, which was higher than that noted for live births (15.8%), providing the evidence that mosaicism may increase the risk of early pregnancy loss [7]. Consistent with the study of Maxwell et al*.*, our study also found a higher mosaicism rate in the case group (17.9% vs. 8.9%), but no significant difference was found, mainly due to the small sample size. Instead of transferring euploid embryos by aCGH as performed in the study of Maxwell et al*.*, embryos were transferred with no CCS results available in our study. Therefore, our study may reflect the true chromosomal status in embryos leading to miscarriage.

However, 5 embryos with the a percentage of mosaicism ranging from 35 to 75% were found in the live birth group, and we observed the presence of a high segmental mosaicism (greater than 50%) in three controls, which arose the question of false-positive predictive value of mosaicism, and the consideration of whether the percentage of mosaicism could be used as a parameter for risk stratification of embryos with mosaicism results. From a biological standpoint, such mosaic levels are influenced by the timing and mechanism of chromosome mis-segregation. Mitotic errors occurring during early postzygotic divisions will spread to a larger proportion of daughter cells [2]. Data from mosaic embryo transfers may represent indirect clinical evidence for self-correction by a mechanism of clonal depletion, which may alter this ratio [28]. Nevertheless, we cannot ignore the information provided by PGT-A, including mosaic level and type, based on the results of 1000 mosaic embryo transfer study [29].

Of note, one chaotic embryo was found in the live-birth group, which was mainly due to artificial inflation of read counts caused by the bias in WGA. Another explanation may involve DNA replication in the biopsied cells. When DNA is replicated unevenly across the genome at S-phase, it could theoretically produce false-positive profiles. Dimitriadou et al*.* showed that single cell segmental aneuploidy detection was compromised by S phase, demonstrating increased false-positive CNV detection and subsequently contributing to reduced predictive power [30]. Fatemi et al*.* showed that one embryo was chaotic in TE biopsy but euploid in ICM [18]. Currently, distinguishing true imbalances from technical artifacts remains a major challenge in the PGT-A field [31].

Furthermore, the potential for mosaic, chaotic, and segmental aneuploidy predictions derived from technical artifacts should be carefully considered. As mentioned above, if no other embryos are available, embryos with segmental abnormality could undergo a second round of biopsy and PGT-A to identify those with increased potential to result in a healthy birth. In addition, we should not rush into the decision of discarding those chaotic embryos caused by technical artifacts. Importantly, transfer of such embryos should be accompanied by genetic counsel with emphasis on prenatal testing, particularly amniocentesis, since fetuses are under unknown risks.

In our study, chromosome abnormalities could not explain the reason for miscarriage in euploid embryos. Other factors, such as repeated induced abortion, sleep deprivation [32], or smoking were also associated with an increased risk of miscarriage [33]. In addition, the resolution of NGS was 5–10 Mb [8], which could not differentiate abnormalities beyond resolution.

The greatest strength of the present study was that the embryos were transferred after PGT-M when no CCS results were available. To compare the clinical outcomes with the PGT-A results, we blindly reanalyzed the reserved MDA products with NGS. This design represents a blinded nonselection study in which blastocysts were biopsied and transferred in the absence of chromosome testing. In this sense, we not only determined the predictive value of euploidy and whole-chromosome aneuploidy in PGT-A, but the findings also may reflect the true chromosomal status in embryos leading to miscarriage and provide evidence that some undetermined reproductive competent embryos labeled abnormal retained the ability to result in a live birth. In addition, a matched case–control study was used to eliminate the confounding effect of age and the embryo culture system on chromosomal abnormalities.

Our study has several limitations. One limitation of this study is that the number of cases analyzed was not sufficiently, and limited data were not powered to determine the predictive values of mosaics, segmental abnormalities, or chaotic verification by NGS. Another limitation is the lack of karyotype analysis of placenta, umbilical cord blood, or products of conception from miscarriages. Only seven of the embryo transfers that resulted in live births were prenatally diagnosed with no abnormal results, and no signs of ﻿morphological abnormalities were noted in any newborn.

## Conclusion

To conclude, greater than half of the miscarriages may be due to chromosomal abnormalities. However, abnormalities, such as segmental aneuploidy and mosaicism, should be managed cautiously given their undermined reproductive potential.

## Data Availability

The datasets used or analysed during the current study is available in https://ngdc.cncb.ac.cn/gsa-human/browse/HRA001819.

## Abbreviations

MDA: Multiple displacement amplification
PGT-Ms: Preimplantation genetic testing for monogenic disorders
PGT-A: Preimplantation genetic testing for aneuploidy screening
CCS: Comprehensive chromosomal screening
NGS: Next-generation sequencing
TE: Trophectoderm
CNV: Copy number variation
ICSI: Intracytoplasmic sperm injection
PCR: Polymerase chain reaction
COS: Controlled ovarian stimulation
WGA: Whole-genome amplification
ICM: Inner cell mass
